# Risk factors and prediction score model for unplanned readmission among neonates with NRDS under one year of age: A retrospective cohort study

**DOI:** 10.3389/fped.2022.964554

**Published:** 2022-10-13

**Authors:** Weihong Yue, Hong Wei, Feng Chen, Xinhong Chen, Zhen-E Xu, Ya Hu

**Affiliations:** ^1^ Department of Neonatology, Children’s Hospital of Chongqing Medical University, Chongqing, China; ^2^ National Clinical Research Center for Child Health and Disorders, Chongqing, China; ^3^ Ministry of Education Key Laboratory of Child Development and Disorders, Chongqing, China; ^4^ Chongqing Key Laboratory of Pediatrics, Chongqing, China

**Keywords:** risk factors, readmission, neonates, neonatal respiratory distress syndrome, unplanned

## Abstract

**Objective:**

This study aimed to analyze the risk factors and establish a prediction score model for unplanned readmission among neonates with neonatal respiratory distress syndrome (NRDS) for respiratory problems under one year of age.

**Methods:**

This retrospective cohort study enrolled 230 neonates with NRDS who were admitted between January 2020 and December 2020. The infants were classified into two subgroups based on whether they were readmitted for respiratory problems under one year of age: readmit group and non-readmit group. Readmission risk factors for NRDS were analyzed by logistic regression and a prediction score model was generated.

**Results:**

Among the 230 enrolled infants, 51 (22%) were readmitted, and 179 (78%) were not readmitted. In univariate analysis, compared with non-readmit group infants, readmit group infants had a significantly younger birth gestational age (31.9 ± 2.3 vs. 32.8 ± 2.5 weeks, *p* = 0.012), lower birth weight (1,713.7 ± 501.3 g vs. 1,946.8 ± 634.4 g, *p* = 0.007), older age at discharge (41.7 vs. 31.7 days, *p* = 0.012), higher proportion of necrotizing enterocolitis (NEC) (31% vs. 16%, *p* = 0.016), higher rate of blood transfusion (39% vs. 25%, *p* = 0.049), higher rate of postnatal dexamethasone (DEX) administration (28% vs. 9.5%, *p* = 0.001), and higher rate of home oxygen therapy (HOT) (57% vs. 34%, *p* = 0.003). Moreover, readmit group infants had significantly longer antibiotic days usage (12.0 vs. 10.0 days, *p* = 0.026) and a longer duration of hospital stay (41.0 vs. 31.0 days, *p* = 0.012) than non-readmit group infants. The multivariate logistic regression analysis showed that taking readmission as a target variable, postnatal DEX administration (OR: 2.689, 95% CI: 1.168–6.189, *p* = 0.020), HOT (OR: 2.071, 95% CI: 1.060–4.046, *p* = 0.033), and NEC (OR: 2.088, 95% CI: 0.995–4.380, *p* = 0.051) could be regarded as risk factors for readmission. A scoring model predicting readmission was administered with a positive predictive value of 0.651 (95% CI: 0.557–0.745, *p* = 0.002), with a sensitivity of 0.412 and a specificity of 0.888 at a cut-off of 3.5 points, which were evaluated on the receiver operating characteristic curve.

**Conclusions:**

Postnatal DEX administration, HOT, and NEC were risk factors for readmission of NRDS. NRDS infants with a predictive score of 3.5 points or more were at high risk for unplanned readmission.

## Introduction

Neonatal respiratory distress syndrome (NRDS) is one of the most common morbidities in premature infants (1–4). The incidence of NRDS increases with decreasing gestational age (GA), and the morbidity in extremely preterm infants (GA < 28 weeks) is almost 93% (5). Although the incidence of NRDS is lower in late preterm and term infants, it still occurs in a significant number from 0.3% to 11% (5). NRDS is still one of the main causes of neonatal respiratory failure and death in neonatal intensive care units at present (1–3).

However, most previous studies on NRDS focused on its onset, severity, management, and resolution (3, 6, 7) or did not focus on readmission. Infants with NRDS are at increased risk for bronchopulmonary dysplasia (BPD), necrotizing enterocolitis (NEC), and neurodevelopmental disabilities, which all subsequently increase the risk of readmitting (8, 9). Unplanned readmission has a great impact on healthcare expenses, hospital stay, and medical quality (10, 11). It will not only result in the separation of mother and infant, which is not conducive to breastfeeding but also increases the risk of pain in infants and affects their growth and development (10).

Although there have been some studies that assessed the association of risk factors with readmission among patients with respiratory distress syndrome in adults (12–14), little attention has been given to unplanned readmission of NRDS in neonates. We found no report on unplanned readmission of NRDS in neonates at present, and the risk factors for readmission among neonates with NRDS remain unclear. Therefore, it is necessary to further identify specific risk factors for unplanned hospital readmission among this particular population to develop quality improvement initiatives and a specific follow-up intervention for high-risk readmission babies.

Our retrospective study was to identify risk factors and establish a prediction score model for unplanned readmission among neonates with NRDS for respiratory problems under one year of age and to provide new directions for clinical practice.

## Materials and methods

### Definitions

(1) The diagnosis of NRDS is based on a clinical picture of an infant with the onset of progressive respiratory failure shortly after birth (manifested by an increase in the work of breathing and an increase in the oxygen requirement), in conjunction with a characteristic chest radiograph (15, 16). Typical imaging findings include a low lung volume and the classic diffuse reticulogranular ground glass appearance with air bronchograms (17). (2) BPD was diagnosed according to the requirement of oxygen supplementation either at 28 days postnatal age or 36 weeks postmenstrual age (18). (3) Sepsis was diagnosed according to the consensus of Chinese experts (2019 version) (19). (4) The diagnosis of bacterial meningitis was based on neonates with positive cerebrospinal fluid (CSF) culture or some cases, although with negative CSF culture, but the initial blood culture grew a pathogen, and the CSF obtained 24–36 h after initiation of antibiotic therapy was abnormal (20, 21). (5) NEC (Bell stage 2 or 3) was defined according to the classification of Bell (22). (6) Shock was diagnosed based on clinical features such as hypotension, decreased peripheral perfusion (including signs of cool extremities, acrocyanosis, pallor, or delayed capillary refill >4 s), or need for the vasoactive drug to maintain blood pressure in the normal range (dopamine >5 μg/kg/min or dobutamine, epinephrine, or norepinephrine at any dose) (23). (7) Persistent pulmonary hypertension and patent ductus arteriosus were diagnosed according to postnatal echocardiography (24). (8) Postnatal dexamethasone (DEX) administration was defined as intravenous administration of dexamethasone according to the Dexamethasone: A Randomized Trial (DART) regimens (0.89 mg/kg for 10 days) for at least one course (25). (9) The definition of home oxygen therapy (HOT) refers to the administration of oxygen *via* nasal cannula in stable preterm infants who do not require a hospital environment (26). In our study, the infant who would initiate HOT should be medically stable, grow well, and have a mean SpO_2_ of 90%–95% without frequent episodes of desaturations. Meanwhile, infants need a low oxygen flow of less than 2 L/min before being discharged home. (10) Gestational diabetes mellitus was defined as diabetes diagnosed during pregnancy (27). (11) Pregnancy-induced hypertension was defined as newly diagnosed hypertension in a pregnant woman after 20 weeks of gestation (28).

### Study design and participants

This retrospective single-center cohort study was conducted in the Department of Neonatology at Children's Hospital of Chongqing Medical University. Our center is a national clinical specialty department that has 300 beds and admits approximately 9,000–10,000 newborns each year. As a pediatric tertiary hospital without a delivery room, all infants were admitted to the NICU from other hospitals. Babies who were diagnosed with NRDS between January 2020 and December 2020 were included in the present study.

Human studies were reviewed and approved by the Clinical Research Ethics Committee of Chongqing Medical University (Registration number: 2022/R/59). The Ethics Committee waived the requirement for informed consent due to the anonymized nature of the data and the scientific purpose of the study.

The study sample consisted of two groups of infants: (1) those that were readmitted for respiratory problems under one year of age (readmit) and (2) those that were not readmitted (non-readmit).

### Inclusion and exclusion criteria

The study sample consisted of newborns admitted to NICU with a diagnosis of NRDS (15, 16), who received standard care and therapy, and discharged (as medically advised), and readmitted or not readmitted for respiratory problems under one year of age. Newborns that were admitted to NICU and received treatment at their birth hospital prior to admission to our NICU were not included in the study sample due to inconsistent treatment protocols and unknown or missing treatment information. The following were the exclusion criteria: (1) congenital heart and lung malformations or clear chromosomal abnormalities, (2) discharged against medical advice during the study, (3) death during the first hospital stay due to a factor other than the respiratory system, (4) readmission as planned, (5) readmission not for respiratory problems, (6) loss to follow-up, and (7) incomplete medical records.

The main outcome variables were readmissions to the hospital at 60-day and under 1 year of age, and death was a secondary outcome among babies who were diagnosed with NRDS.

The standard care and therapy for NRDS after admission to our NICU mainly include the following process: (1) Respiratory support including invasive or noninvasive mechanical ventilation (MV); (2) Pulmonary surfactant drug therapy: Medication timing: Use of therapeutic pulmonary surfactant as early as possible in the early stage of the disease (generally 2 h). The dosage was 200 mg/kg of pig lung surfactant or 100 mg/kg of bovine lung surfactant; dosing times: once for mild cases, multiple dosing is required for severe cases, and dosing as needed.

### Data collection

Relevant information was retrospectively collected by trained staff using standardized data collection and quality control procedures from the medical data platform of Children's Hospital of Chongqing Medical University, including demographics [gender, birth gestational age, gestational age at discharge, birth weight (BW), discharge weight, admission age, discharge age, and single or multiple births], high-risk perinatal factors [cesarean section, pregnancy-induced hypertension, gestational diabetes mellitus, premature rupture of membranes (PROM), placental abruption, intrauterine distress and nature of amniotic fluid], basic vital signs (heart rate, respiratory rate, temperature), blood gas findings (pH, PCO_2_, PO_2_, HCO_3_, BE, SpO_2_, serum lactic acid, and blood sugar), management during hospitalization [mechanical ventilation, enteral feeds (including initiation time of enteral feeds, milk volume on the discharge day, proportion of nasal feeds), nitric oxide inhalation, postnatal DEX administration, packed red blood cell transfusion, peripherally inserted central catheter (PICC), and antibiotic], neonatal complications or comorbidities (bacterial meningitis, retinopathy of prematurity, patent ductus arteriosus, persistent pulmonary hypertension, NEC, sepsis, shock, pulmonary hemorrhage, pneumothorax, and BPD), length of hospital stay and hospitalization cost with Renminbi (RMB, China's currency).

A phone follow-up was conducted to document the outcomes of readmission and death for all those that met the inclusion criteria. Those lost to follow-up could not be contacted due to a disconnected phone number given to the NICU at admission.

### Statistical analysis

All data were analyzed by SPSS 26.0 software (IBM, Armonk, New York). Normally distributed continuous data were described as the mean ± standard deviation (M ± SD) and tested by Student's *t*-test. Skewed data were described as the median and interquartile range (IQR) and analyzed by the Mann–Whitney *U* test. Categorical data are shown as percentages and were analyzed by the chi-square test or Fisher's exact test. To determine potential risk factors for readmission, multivariate logistic regression was performed. Prior to model development, clinical variables (having a univariate result of *p*-value <0.05) were tested for multicollinearity using variance inflation factor (VIF). A variable was removed having a VIF greater than 2.5 and VIF was repeated until all variables had a VIF less than or equal to 2.5. Two of the continuous variables were converted to binary: (1) very low BW and extremely low infants (BW < 1,500 g) were labeled “1” and the others (BW ≥ 1,500 g) were “0”; and (2) antibiotic days cut-off value was determined by receiver operating characteristic (ROC) curve [8.5 days; area under the curve (AUC) = 0. 602, 95% CI: 0.519–0.686] in which less than 8.5 days was labeled “1” and the others (time ≥ 8.5 days) were “0”. A scoring model predicting readmission was developed from the significant regression coefficients. ROC curve and AUC were used to measure and evaluate the predictive values of the model. A *p*-value <0.05 was considered statistically significant.

## Results

### Demographics and clinical characteristics of the two groups

**Figure 1:**
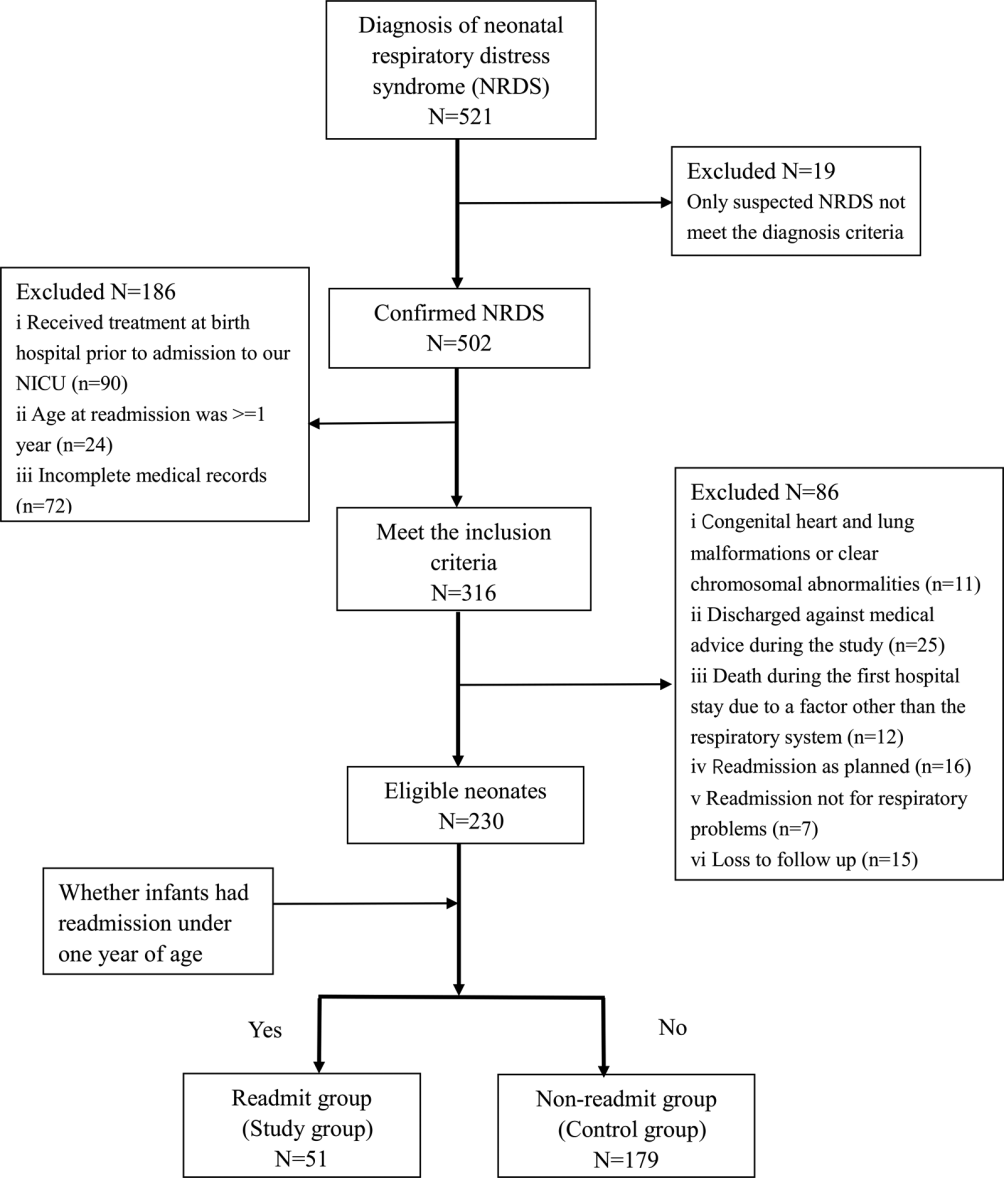
Patient selection flowchart.

Figure 1 displays a flow diagram of inclusion and exclusion criteria of the study sample. A total of 521 infants with confirmed NRDS were admitted to our NICU between January 2020 and December 2020. Among them, 316 (61%) infants met the inclusion criteria, but 86 cases were further excluded based on the exclusion criteria. Among excluded cases, there were 25 (7.9%) infants discharged against medical advice from the NICU in our study and excluded finally. Meanwhile, there were 15 families lost to follow-up who could not be contacted due to a disconnected phone number given to the NICU at admission, and also could not be found any of their second or more readmission records in our hospital. Therefore, the study sample was 230 neonates with NRDS of which there were 51 (22%) had readmission to the hospital for respiratory problems under 1 year of age, and 179 (78%) did not have readmission. Eleven of the neonates (4.8%) were full-term (birth gestational age ≥37 weeks), two-thirds were male, and the median age at admission to the NICU was 1 h (range 1–2 h).

Infants in the Readmit group had a significantly smaller birth gestational age, lower birth weight, and older age at discharge than infants in the non-Readmit group (31.9 ± 2.3 vs. 32.8 ± 2.5 weeks, *p* = 0.012; 1,713.7 ± 501.3 g vs. 1,946.8 ± 634.4 g, *p* = 0.007; 41.7 vs. 31.7 days, *p* = 0.012, respectively). Moreover, the proportion of intrauterine distress was slightly higher in the readmit group than in the non-readmit group (26% vs. 15%, *p* = 0.066). In contrast, there were no differences in gender, collected gestational age at discharge, admission age, basic vital signs (heart rate, respiratory rate, and temperature) at admission or discharge, or high-risk perinatal factors, as shown in Table 1.

**Table 1:** Demographics and clinical characteristics of the two groups.

Variables	Total (*N* = 230)	Readmit group (*N* = 51)	Non-readmit group (*N* = 179)	*p*-value
Demographics				
Male gender, *n* (%)	154 (67)	36 (70)	118 (66)	0.532
Birth GA (weeks)^a^	32.6 ± 2.5	31.9 ± 2.3	32.8 ± 2.5	0.012
Collected GA at discharge (weeks)^a^	38.0 ± 1.8	38.3 ± 2.0	38.0 ± 1.7	0.350
Age at admission (hours)^b^	1.0 (1.0, 2.0)	1.0 (1.0, 2.0)	1.0 (1.0, 2.0)	0.616
Age at discharge (days)^b^	34.6 (19.7, 51.9)	41.7 (27.7, 63.7)	31.7 (18.6, 51.4)	0.012
BW (g)^a^	1,895.1 ± 614.1	1,713.7 ± 501.3	1,946.8 ± 634.4	0.007
Weight at discharge (g)^a^	2,553.4 ± 401.4	2,515.7 ± 373.4	2,564.1 ± 409.4	0.448
Full-term infants, *n* (%)	11 (4.8)	1 (2.0)	10 (5.6)	0.463^c^
Multiple births, *n* (%)	78 (34)	19 (37)	59 (33)	0.568
Basic vital signs				
Heart rate at admission (beats/min)^a^	136.6 ± 12.1	136.6 ± 11.0	136.5 ± 12.5	0.962
Respiratory rate at admission (beats/min)^a^	55.3 ± 6.5	55.8 ± 6.2	55.2 ± 6.6	0.528
Temperature at admission (°C)^a^	36.1 ± 0.5	36.1 ± 0.5	36.2 ± 0.6	0.516
Heart rate at discharge (beats/min)^a^	137.3 ± 9.9	137.7 ± 8.5	137.2 ± 10.3	0.730
Respiratory rate at discharge (beats/min)^a^	45.8 ± 6.9	45.9 ± 2.3	45.7 ± 7.8	0.859
Temperature at discharge (°C)^a^	36.7 ± 0.2	36.7 ± 0.1	36.7 ± 0.2	0.406
High-risk perinatal factors				
Cesarean section, *n* (%)	198 (86)	46 (90)	152 (85)	0.336
Gestational diabetes mellitus, *n* (%)	57 (25)	9 (18)	48 (27)	0.181
Pregnancy-induced hypertension, *n* (%)	20 (8.7)	5 (9.8)	15 (8.4)	0.779^c^
Nature of amniotic fluid, *n* (%)				
Meconium stained	16 (7.0)	4 (7.8)	12 (6.7)	0.870
Hemorrhagic	6 (2.6)	2 (3.9)	4 (2.2)	
Normal	186 (81)	41 (80)	145 (81)	
Unknown	22 (9.6)	4 (7.8)	18 (10)	
PROM > 18 h, *n* (%)	70 (30)	17 (33)	53 (30)	0.610
Intrauterine distress, *n* (%)	39 (17)	13 (26)	26 (15)	0.066
Placental abruption, *n* (%)	13 (5.7)	4 (7.8)	9 (5.0)	0.492

^a^ Mean and standard deviation.

^b^ Median and interquartile range.

^c^ Fisher's exact test.

GA, gestational age; BW, birth weight; PROM, premature rupture of membranes.

### Complications, comorbidities, and blood gas findings of the two groups

The incidence of NEC significantly increased in the readmit group compared with the non-readmit group (31% vs. 16%, *p* = 0.016). Infants in the readmit group had slightly higher morbidity of sepsis and BPD than infants in the non-readmit group (60.8% vs. 49.6%, *p* = 0.069; 41.2% vs. 27.4%, *p* = 0.059, respectively). On the other hand, there were no differences in morbidity of shock, bacterial meningitis, retinopathy of prematurity, patent ductus arteriosus, persistent pulmonary hypertension of the newborn, pulmonary hemorrhage, or pneumothorax, as shown in Table 2.

**Table 2:** Complication, comorbidity, and blood gas of the two groups.

Variables	Total (*N* = 230)	Readmit group (*N* = 51)	Non-readmit group (*N* = 179)	*p*-value
Complication/comorbidity				
Shock, *n* (%)	17 (7.4)	3 (5.9)	14 (7.8)	0.770
Sepsis, *n* (%)	114 (50)	31 (61)	83 (46)	0.069
NEC, *n* (%)	45 (20)	16 (31)	29 (16)	0.016
Bacterial meningitis, *n* (%)	11 (4.8)	4 (7.8)	7 (3.9)	0.267
Retinopathy of prematurity, *n* (%)	31 (14)	8 (16)	23 (13)	0.601
Patent ductus arteriosus, *n* (%)	128 (56)	32 (63)	96 (54)	0.248
Persistent pulmonary hypertension, *n* (%)	91 (40)	23 (45)	68 (38)	0.360
Pulmonary hemorrhage, *n* (%)	27 (12)	8 (16)	19 (11)	0.321
Pneumothorax, *n* (%)	5 (2.2)	1 (2.0)	4 (2.2)	1.000^a^
BPD, *n* (%)	70 (30)	21 (41)	49 (27)	0.059
Blood gas findings				
The first results after admission				
pH^b^	7.3 ± 0.1	7.3 ± 0.1	7.3 ± 0.1	0.939
PCO_2_ (mmol/L)^c^	45.5 (36.0, 57.0)	47.0 (36.0, 62.0)	45.0 (36.0, 56.0)	0.586
PO_2_ (mmol/L)^c^	84.0 (56.5, 131.3)	81.0 (58.0, 140.0)	86.0 (55.0, 131.0)	0.892
SpO_2_ (%)^c^	95.0 (83.8, 99.0)	96.0 (84.0, 99.0)	95.0 (83.0, 99.0)	0.458
HCO_3_ (mmol/L)^c^	22.3 (20.0, 25.0)	23.7 (20.3, 25.4)	22.1 (19.9, 25.0)	0.180
Base deficit (mmol/L)^c^	−4.1 (−6.2, −2.2)	−3.4 (−5.6, −1.4)	−4.4 (−6.2, −2.3)	0.124
Blood sugar (mmol/L)^b^	4.8 ± 2.2	4.6 ± 2.4	4.9 ± 2.1	0.451
Blood lactic acid (mmol/L)^b^	2.4 ± 1.5	2.2 ± 1.7	2.5 ± 1.5	0.250
The last results prior to discharge^d^				
pH^b^	7.4 ± 0.1	7.4 ± 0.1	7.4 ± 0.1.	0.662
PCO_2_ (mmol/L)^c^	40.0 (34.0, 46.0)	40.0 (34.0, 47.5)	40.0 (33.0, 46.0)	0.567
PO_2_ (mmol/L)^c^	75.0 (59.0, 98.0)	73.0 (65.0, 102.0)	75.5 (57.8, 97.3)	0.471
SpO_2_ (%)^c^	95.0 (90.0, 98.0)	95.0 (89.0, 98.0)	95.0 (91.5, 98.0)	0.857
HCO_3_ (mmol/L)^c^	23.1 (20.2, 26.4)	23.7 (20.8, 26.9)	22.8 (20.1, 26.4)	0.369
Base deficit (mmol/L)^c^	−2.4 (−4.8, 1.0)	−2.0 (−4.1, 2.1)	−2.8 (−5.1, 0.6)	0.310
Blood sugar (mmol/L)^b^	4.4 ± 1.6	4.4 ± 1.4	4.4 ± 1.6	0.922
Blood lactic acid (mmol/L)^b^	1.8 ± 1.1	1.7 ± 0.9	1.8 ± 1.2	0.673

^a^ Fisher's exact test.

^b^ Mean and standard deviation.

^c^ Median and interquartile range.

^d^ Only 139 (60.4%) infants (33 cases in the Readmit group and 106 cases in the non-Readmit group) had blood gas data obtained during the 3 days prior to discharge and were analyzed for this variable.

NEC, necrotizing enterocolitis; BPD, bronchopulmonary dysplasia.

The blood gas data, including pH, PCO_2_, PO_2_, HCO_3_, BE, SpO_2_, serum lactic acid, and blood sugar at admission or before discharge, were not significantly different between the two groups (all *p* > 0.05) (Table 2).

### Management of the two groups

The readmit group needed longer courses of antibiotics, more postnatal DEX administration, and more packed red blood cell transfusions than the non-readmit group (12.0 vs. 10.0 days, *p* = 0.026; 27.5% vs. 9.5%, *p* = 0.001; 39% vs. 25%, *p* = 0.049, respectively). Meanwhile, there were significantly increased requirements for HOT among infants in the readmit group at discharge compared to infants in the non-readmit group (57% vs. 34%, *p* = 0.003). In contrast, there were no differences in terms of the duration of total MV, invasive MV or noninvasive MV, enteral feeds (including initiation time of enteral feeds, milk volume on the first day and the discharge day, the proportion of nasal feeds), proportion of nitric oxide inhalation, and duration of PICC. Moreover, the median duration and cost of hospital stay were longer or higher in the readmit group than in the non-readmit group (41.0 vs. 31.0 days, *p* = 0.012; RMB 198,586.1 vs. 158,293.0, *p* = 0.009) (Table 3).

**Table 3:** Management of the two groups.

Variables	Total (*N* = 230)	Readmit group (*N* = 51)	Non-readmit group (*N* = 179)	*p*-value
Duration of hospital stay (days)^a^	34.0 (19.0, 51.3)	41.0 (27.0, 63.0)	31.0 (17.0, 50.0)	0.012
Hospitalization cost (RMB)^a^	190,485.6 (130,186.3, 273,493.4)	198,586.1 (158,293.0, 331,112.4)	180,042.3 (123,909.8, 256,267.0)	0.009
Respiratory support				
Duration of invasive MV (days)^a^	5.0 (2.0, 7.0)	6.0 (4.0, 11.0)	4.5 (2.0, 6.3)	0.138
Duration of noninvasive MV (days)^a^	6.0 (3.0, 15.0)	9.0 (4.0, 27.0)	6.0 (3.0, 14.0)	0.525
Total duration of MV (days)^a^	9.0 (5.0, 20.0)	12.0 (5.0, 38.0)	9.0 (5.0, 19.0)	0.306
Duration off oxygen therapy prior to discharge (days)^a^	4.0 (0.0, 8.0)	3.5 (0.0, 8.5)	4.0 (0.0, 8.0)	0.993
Invasive MV, *n* (%)	121 (53)	27 (53)	94 (53)	0.957
HOT, *n* (%)	89 (39)	29 (57)	60 (34)	0.003
Antibiotic therapy				
Antibiotic days usage (days)^a^	11.0 (5.8, 22.3)	12.0 (8.0, 28.0)	10.0 (5.0, 20.0)	0.026
Duration off antibiotic prior to discharge (days)^a^	13.0 (6.0, 24.0)	16.0 (6.0, 27.0)	12.0 (6.0, 23.0)	0.466
Enteral feeds				
Initiation time of enteral feeds (days)^a^	1.0 (1.0, 1.0)	1.0 (1.0, 1.0)	1.0 (1.0, 2.0)	0.453
Milk volume on the discharge day, (ml/kg. day)^a^	153.1 (135.0, 166.8)	157.4 (140.0, 173.4)	151.5 (134.0, 164.7)	0.115
Complete or partial nasal feeds at discharge, *n* (%)	8 (3.5)	3 (5.9)	5 (2.8)	0.380^b^
Adjuvant therapy				
Duration of PICC (days)^a^	12.0 (0.0, 26.3)	16.0 (0.0, 31.0)	11.0 (0.0, 24.0)	0.165
Packed red blood cell transfusions once at least, *n* (%)	65 (28)	20 (39)	45 (25)	0.049
Nitric oxide inhalation for 24 h at least, *n* (%)	5 (2.2)	1 (2.0)	4 (2.2)	1.000^b^
Postnatal dexamethasone administration, *n* (%)	31 (14)	14 (28)	17 (9.5)	0.001

^a^ Median and interquartile range.

^b^ Fisher's exact test.

PICC, peripherally inserted central catheter; HOT, home oxygen therapy; MV, mechanical ventilation.

### Logistic regression analysis of risk factors associated with readmission in neonates with NRDS

Seven variables were tested in the multivariate logistic model (BW, NEC, packed red blood cell transfusions, postnatal DEX administration, HOT, antibiotic days usage, and gender) in the first step, and four values were excluded with a *p* > 0.05 and an OR around 1.0 which means these had no value in it (Table 4A). “Antibiotic days usage” was discovered as an adjustor which was attenuating NEC when removing it in model development. After further analysis, the significant risk factors for readmission were postnatal DEX administration (OR: 2.689, 95% CI: 1.168–6.189, *p* = 0.020), HOT (OR: 2.071, 95% CI: 1.060–4.046, *p* = 0.033), and NEC (OR: 2.088, 95% CI: 0.995–4.380, *p* = 0.051). The logistic regression equation was logistic (readmission) = −1.923 + 0.989 (postnatal DEX administration) + 0.728 (HOT) + 0.736 (NEC) (*X*^2^ = 18.312, *p* = 0.000), with 79.6% correct predictions, as shown in Table 4B.

**Table 4:** Logistic regression analysis of risk factors associated with readmission in neonates with NRDS.

Independent variables	*β*	SE	Wald	*p*	OR	95% CI
(A) Primary model including all values in the first step						
Postnatal DEX administration (Yes = 1 No = 0)	1.002	0.484	4.289	0.038	2.723	1.055 to 7.029
HOT (Yes = 1 No = 0)	0.746	0.366	4.158	0.041	2.108	1.029 to 4.316
NEC (Yes = 1 No = 0)	0.773	0.393	3.859	0.049	2.165	1.002 to 4.681
Gender (Male = 1 Female = 0)	−0.282	0.369	0.582	0.446	0.754	0.366 to 1.556
Packed red blood cell transfusions (Yes = 1 No = 0)	0.342	0.393	0.757	0.384	1.408	0.651 to 3.042
Birth weight < 1,500 g (Yes = 1 No = 0)	0.000	0.000	0.284	0.594	1.000	0.999 to 1.001
Antibiotic days usage <8.5 days (Yes = 1 No = 0)	−0.015	0.013	1.266	0.260	0.985	0.961 to 1.011
Constant	−1.363	0.856	2.535	0.111	0.256	
(B) Final model removed values without statistically significant in the last step						
Postnatal DEX administration (Yes = 1 No = 0)	0.989	0.425	5.406	0.020	2.689	1.168 to 6.189
HOT (Yes = 1 No = 0)	0.728	0.342	4.540	0.033	2.071	1.060 to 4.046
NEC (Yes = 1 No = 0)	0.736	0.378	3.793	0.051	2.088	0.995 to 4.380
Constant	−1.923	0.256	56.256	0.000	0.146	

NEC, necrotizing enterocolitis; HOT, home oxygen therapy; DEX, dexamethasone.

### Risk score system for readmission in neonates with NRDS

Table 5 displays the results of the risk score calculation for the three risk factors of postnatal DEX administration, HOT, and NEC. A score of “0” point was assigned when risk factors did not occur. Two points were assigned to HOT which means a one-point equal regression coefficient of 0.364 (=0.728/2). Three points were assigned to postnatal DEX administration (regression coefficient of 0.989/0.364 = 2.717). Two points were assigned to NEC (regression coefficient of 0.736/0.364 = 2.022).

**Table 5:** Risk score system for readmission in neonates with NRDS

Risk factors	β	Rank	Category	Score
Postnatal DEX administration	0.989	No	0	0
		Yes	1	3
HOT	0.728	No	0	0
		Yes	1	2
NEC	0.736	No	0	0
		Yes	1	2
Score maximum				7

NEC, necrotizing enterocolitis; HOT, home oxygen therapy; DEX, dexamethasone.

**Figure 2:**
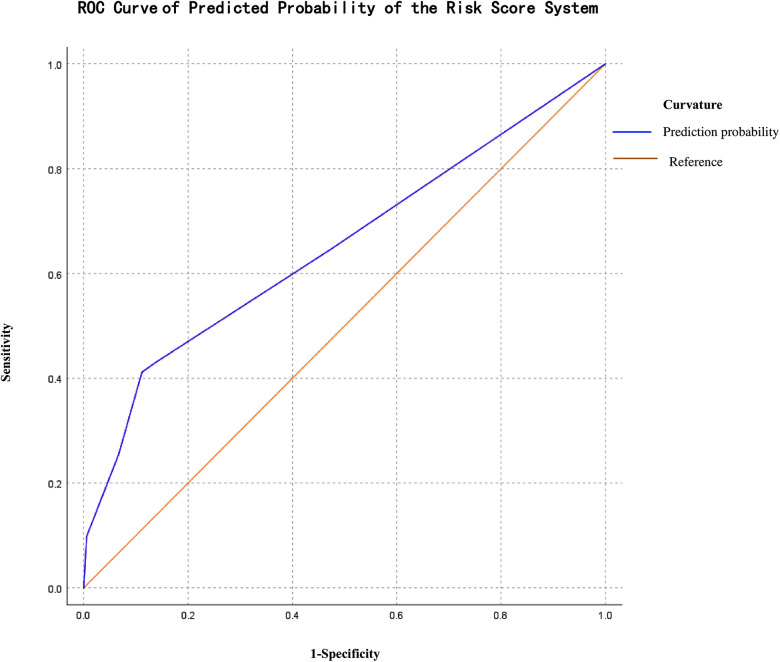
ROC curve of the logistic predicted probability of the risk score system regression analysis.

The median readmission risk factor scores in the readmit group compared with in the non-readmit group were significantly higher (2 vs. 0 points, *p* = 0.000). The positive predictive value for readmission among NRDS was 0.651 (95% CI: 0.557–0.745, *p* = 0.002), with a sensitivity of 0.412 and a specificity of 0.888 at a cut-off of 3.5 points, which were evaluated on the ROC curve (Figure 2). To apply this model, all cases were further divided into two groups: the high-risk Readmit group (*n* = 41) enrolled infants with a score of 3.5 points or more including 21 real readmission babies, and the low-risk Readmit group (*n* = 189) enrolled those with a score less than 3.5 points including 30 real readmission babies. The high-risk Readmit group had a significantly higher readmission rate than the low-risk Readmit group (51% vs. 16%, *p* = 0.000).

### Analysis of information in the 51 neonates with readmission

Among the 51 cases with readmission, 16 (31%) cases developed respiratory failure and needed ventilator assistance after readmission, while all recovered and were discharged. No death existed at 60-day or under 1 year of age among neonates with NRDS who were discharged home based on doctors' decisions in our cohort. The median time and age of readmission were 41.0 days (range: 7–144 ) and 82.0 days (range: 57.0–198.0 days), respectively. The readmission rate was much higher within 60 days after the first discharge than over 60 days (61% vs. 39%, *p* = 0.029).

## Discussion

NRDS is still an important disease affecting infants in neonatal intensive care units (1–3), and even after being successfully cured and discharged, some infants with NRDS may still be at high risk for readmission due to lung problems in the first year of life. The results of our study showed that the unplanned readmission rate was 22% in NRDS infants who received follow-up, which was similar to the extremely low-GA neonates reported before (21%) (29). Our study confirmed that the most common risk factors for readmission in neonates with NRDS were postnatal DEX administration, and HOT, which were very different from previous studies in adults with RDS (12–14), whose readmission was mainly for infection, gastrointestinal, and cardiovascular reasons.

Infants born at lower BW and earlier GA were at a higher risk of readmission (29). In our study, the infants who were more likely to be readmitted had lower birth GA (31.9 weeks, *p* = 0.012) and BW (1.7 ± 0.5 kg, *p* = 0.007), which is consistent with the findings of Pattnaik et al. (29). However, in the multivariate analysis, therapeutic treatments were predictive of readmission than these demographic characteristics.

Despite advances in the treatment of NRDS, including the use of antenatal corticosteroids, exogenous surfactants, and more noninvasive respiratory support, BPD remains a major complication of neonates with NRDS (8, 9, 30). Although BPD was not proven to be a dependent risk factor for readmission in our study, the mean incidence of BPD was 30% and slightly higher in the Readmit group (41%), which increased the proportion of usage of postnatal DEX. The risks vs. benefits of systemic corticosteroids for BPD treatment are not clearly established (31), while evidence still exists for using DEX. In our NICU, systemic corticosteroids are mainly chosen for infants with serious BPD based on a sufficient assessment by clinicians. We found that infants who received systemic DEX therapy (0.89 mg/kg for 10 days based on the DART regimens for at least one course) during hospitalization were associated with a near 2.7 higher odds for readmission compared with those who did not. The adverse effects of corticosteroids, mainly infections and immunosuppression, have been well described (32). Previous studies have shown that there is a linear increase in the risk with the dose and duration of glucocorticoids; often, moderate- to high-dose use of glucocorticoids may significantly increase the risk of infections (33). Several other factors may influence the adverse effects of glucocorticoids, such as age, severity and nature of the underlying disease, and poor nutritional status (32, 33). In our NICU, the DART regimen for BPD was low-dose use of glucocorticoids. Due to young age, low weight and poor lung function affected by NRDS in the neonatal period, even a low dose of glucocorticoids may also lead to an increase in the risk of common mild infections as well as serious life-threatening infections. Our findings have implications for clinical practice to avoid the nonbeneficial use of corticosteroids and limiting what is necessary would be a benefit to reducing readmission.

The practice of delivering HOT to infants is now firmly established in many centers, and BPD was found to have the highest proportion among infants receiving HOT (34). However, the rates of HOT vary between NICUs due to different policies regarding supplemental oxygen use (34–36). In our NICU, when infants are medically stable, growing well, having a mean SpO_2_ of 90%–95% without frequent episodes of desaturations, and only need a low oxygen flow of less than 2 L/min, HOT is commonly prescribed by clinicians mainly based on their clinical experience for them on discharge. However, we found that infants who needed HOT at discharge had a near 2.1 higher odds for readmission than those who did not, which is consistent with DeMauro et al. (34). However, our result was contrary to Lagatta et al. (35), who did not find an association between home oxygen use and 1-year readmission in infants born preterm with BPD discharged (35). This could stem from differences in study populations between our study and Lagatta's. We enrolled infants who had NRDS; only 30% of infants among them had BPD, and the mean birth GA of infants was 32.6 ± 2.5 weeks. In contrast, they enrolled those who had BPD, and their birth GA was less than 32 weeks. Another reason may be due to different clinical experiences with clinical illness and family or community resources impacting the decision in ours and their NICU to use discharge medical equipment such as home oxygen. Thus, we consider that infants who only need a lower oxygen flow <0.5 L/min but can maintain a higher target SpO_2_ of 93%–95% (36), may be safer to receive HOT at discharge and the outpatient follow-up that can reduce readmission risk. Thus, more attention should be given to clinicians to strictly make a decision regarding infants' discharge when they present relatively stable but still need oxygen supplementation.

NEC was more common in Readmit group infants than non-Readmit group infants (31% vs. 16%, *p* = 0.016) with an OR similar to DEX and HOT. When babies developed NEC, why they may be at a higher risk of readmission was unclear. We speculated that it might be related to the nutritional status of newborns with NEC based on the research of Yoo et al. (37). They discovered that nutritional status is closely related to the prognosis of ARDS in adult, and malnutrition may increase the incidence of adverse events (37). The principle of treatment requires fasting for babies diagnosed with NEC for a relatively long time (22), which delays the time of enteral feeding and often remains secondary nutritional problems. Meanwhile, due to impaired intestinal function, and more severity of disease of infants with NRDS complicated NEC, they are more likely to be readmitted.

Moreover, readmit group infants had a longer duration of hospital stay and a higher cost of hospitalization, which was in agreement with the results of Mourani et al. (38). Mourani et al. reported that ICU readmission was more common among increasing durations of birth hospitalization (38). However, our result differs from the study by Harron et al. (39). They found that a longer length of hospital stay was associated with a reduced risk of readmission, but only for late preterm vaginal births (34–36 completed weeks' gestation) (39). We focused on respiratory readmissions under one year of age in our study, rather than all readmissions within 30 days of postnatal discharge in the study of Harron et al. One possible reason for this is that readmitted infants who need longer hospital stays often present that they are unstable and need more time to recover their function of respiratory organs from NRDS, which increases the cost for medical care and therapy due to their severity of the primary disease.

One method for quantifying readmission risk is to develop predictive models using logistic regression. Wu et al. (40) established a scoring system for chronic obstructive pulmonary disease readmission, and Li et al. (41) also developed a mortality prediction scoring system for neonates with pulmonary hemorrhage. However, as we know, no model related to clinical risk factors for readmission among infants with NRDS has been generated at present. In our study, we established a risk score system with a positive predictive value of 0.651 (95% CI: 0.557–0.745, *p* = 0.002), with a sensitivity of 0.412 and a specificity of 0.888 at a cut-off of 3.5 points for readmission caused by NRDS according to logistic regression. Our results indicated that this risk score system has a low value for prediction based on the diagnostic value of AUC (42), which is 0.5–0.7. The high-risk readmit group with a score of 3.5 or more was proven to have a significantly higher readmission rate than the low-risk readmit group. Thus, infants with high scores should be given individual post-discharge care and follow-up for readmission risk. Meanwhile, we must emphasize that the value of this predictive score model was not intended to limit care for the low-scored infant for its low value for prediction.

According to our findings, among the 51 readmission patients, some suffered from respiratory failure but recovered if they could be treated in time after readmission, which is most likely to occur within 60 days after discharge. Thus, we should be vigilant in the long-term respiratory management of infants with NRDS who are at high risk for readmission to avoid life-threatening secondary respiratory failure.

We also compared other factors, such as basic vital signs, high-risk perinatal factors, and blood gas results. However, our findings demonstrated no obvious correlations between these factors and the occurrence of readmission in neonates with NRDS.

The main strength of our study is that our findings may be the first report focused on the risk factors for unplanned readmission among neonates with NRDS, which has important value for clinical practice. In this study, we found potential independent risk factors associated with readmission and demonstrated a predictive model to examine the risks of readmission among infants with NRDS under 1 year of age.

This study has some limitations. First, this is a retrospective series focused on hospitalized patients and inevitably leads to the loss of some clinical and follow-up data. Second, we only included patients whose first admission was to our hospital or whose cause of readmission was respiratory problems. The cases that had been admitted to other hospitals or readmission for other causes may need to be further investigated in the future. Another limitation is that we could not obtain most socioeconomic variables such as babies' parents whether living in rural areas for we did not design it when we collected the data. The gestation age in our study is very narrow—basically 32 weeks and there only 2.2% of babies with gestation age <28 weeks, which is a limitation to our study relative to other countries such as the United States range of 22–38 weeks for the actual differences in birth groups in different regions.

## Conclusion

Postnatal DEX administration, HOT, and NEC were risk factors for readmission of NRDS. NRDS infants with a predictive score of 3.5 points or more were at high risk for unplanned readmission.

## Data Availability

The original contributions presented in the study are included in the article/Supplementary Material, further inquiries can be directed to the corresponding author.

## References

[1] Rijal P Shrestha M. Scenario of neonatal respiratory distress in tertiary hospital. J Nepal Health Res Counc. (2018) 16(2):131–5. PMID: 29983424

[2] Elfarargy MS Al-Ashmawy GM Abu-Risha S Khattab H. Novel predictor markers for early differentiation between transient tachypnea of newborn and respiratory distress syndrome in neonates. Int J Immunopathol Pharmacol. (2021) 35:20587384211000554. 10.1177/20587384211000554 33722097 PMC7970176

[3] Jing L Yun S Dong J Zheng T Li J Lu L Clinical characteristics, diagnosis and management of respiratory distress syndrome in full-term neonates. Chin Med J. (2010) 123(19):2640–4. 21034645

[4] Stoll BJ Hansen NI Bell EF Shankaran S Laptook AR Walsh MC Neonatal outcomes of extremely preterm infants from the NICHD neonatal research network. Pediatrics. (2010) 126:443. 10.1542/peds.2009-2959 20732945 PMC2982806

[5] Consortium on Safe Labor, Hibbard JU Wilkins I Sun L Gregory K Haberman S Respiratory morbidity in late preterm births. JAMA. (2010) 304:419. 10.1001/jama.2010.1015 20664042 PMC4146396

[6] Liszewski MC Stanescu AL Phillips GS Lee EY. Respiratory distress in neonates: underlying causes and current imaging assessment. Radiol Clin North Am. (2017) 55(4):629–44. 10.1016/j.rcl.2017.02.006 28601172

[7] Li Y Zhang C Zhang D. Cesarean section and the risk of neonatal respiratory distress syndrome: a meta-analysis. Arch Gynecol Obstet. (2019) 300(3):503–17. 10.1007/s00404-019-05208-7 31187205

[8] Elder DE Hagan R Evans SF Benninger HR French NP. Hospital admissions in the first year of life in very preterm infants. J Paediatr Child Health. (1999) 35(02):145–50. 10.1046/j.1440-1754.1999.00308.x 10365350

[9] Morris BH Gard CC Kennedy K; NICHD Neonatal Research Network. Rehospitalization of extremely low birth weight (ELBW) infants: are there racial/ethnic disparities? J Perinatol. (2005) 25(10):656–63. 10.1038/sj.jp.7211361 16107873

[10] Landrum L Weinrich S. Readmission data for outcomes measurement: identifying and strengthening the empirical base. Qual Manag Health Care. (2006) 15(2):83–95. 10.1097/00019514-200604000-00003 16622357

[11] Jencks SF Williams MV Coleman EA. Rehospitalizations among patients in the Medicare fee-for-service program. N Engl J Med. (2009) 360(14):1418–28. 10.1056/NEJMsa0803563 19339721

[12] Siuba MT Sadana D Gadre S Bruckman D Duggal A. Acute respiratory distress syndrome readmissions: a nationwide cross-sectional analysis of epidemiology and costs of care. PLoS One. (2022) 17(1):e0263000. 10.1371/journal.pone.0263000 35077505 PMC8789165

[13] Shah H Mansuri U Pagad S Adupa R Singh J Tun K Rate and modifiable predictors of 30-day readmission in patients with acute respiratory distress syndrome in the United States. Cureus. (2020) 12(6):e8922. 32760623 10.7759/cureus.8922 PMC7392362

[14] Wozniak AW Pfoh ER Dinglas VD Pronovost PJ Needham DM Colantuoni E Readmission and subsequent decline in long-term survivors of acute respiratory distress syndrome. Am J Crit Care. (2019) 28(1):76–80. 10.4037/ajcc2019580 30600230

[15] Perepelitsa SA Golubev AM Moroz VV. Neonatal respiratory distress syndrome: early diagnosis, prevention, and treatment. General Reanimatology. (2012) 8(4):95. 10.15360/1813-9779-2012-4-95

[16] Reuter S,Moser C, Baack M. Respiratory distress in the newborn. Pediatr Rev. (2014) 35(10):417–28. 10.1542/pir.35-10-417 PMC4533247 25274969

[17] Kramer B. The respiratory distress syndrome in preterm infants: physiology, prophylaxis and new therapeutic approaches. Intensivmed Notfmed. (2007) 44(44):403–8. 10.1007/s00390-007-0809-3

[18] Poindexter BB Feng R Schmidt B Aschner J Ballard R Hamvas A Comparisons and limitations of current definitions of bronchopulmonary dysplasia for the prematurity and respiratory outcomes program. Ann Am Thorac Soc. (2015) 12:1822. 10.1513/AnnalsATS.201504-218OC 26397992 PMC4722827

[19] The Subspecialty Group of Neonatology, the Society of Pediatric, Chinese Medical Association, Professional Committee of Infectious Disease, Neonatology Society, Chinese Medical Doctor Association. Expert consensus on the diagnosis and management of neonatal sepsis (version 2019). Chin J Pediatr. (2019) 57:252–7. 10.3760/cma.j.issn.0578-1310.2019.04.005 30934196

[20] Klinger G Chin CN Beyene J Perlman M. Predicting the outcome of neonatal bacterial meningitis. Pediatrics. (2000) 106:477. 10.1542/peds.106.3.477 10969090

[21] Ouchenir L Renaud C Khan S Bitnun A Boisvert A McDonald J The epidemiology, management, and outcomes of bacterial meningitis in infants. Pediatrics. (2017) 140:e20170476. 10.1542/peds.2017-0476 28600447

[22] Bell MJ Ternberg JL Feigin RD Keating JP Marshall R Barton L Neonatal necrotizing enterocolitis. Therapeutic decisions based upon clinical staging. Ann Surg. (1978) 187(1):1. 10.1097/00000658-197801000-00001 413500 PMC1396409

[23] Davis AL Carcillo JA Aneja RK Deymann AJ Lin JC Nguyen TC The American college of critical care medicine clinical practice parameters for hemodynamic support of pediatric and neonatal septic shock: executive summary. Crit Care Med. (2017) 45:1061. 10.1097/CCM.0000000000002425 28509730

[24] Dhillon R. The management of neonatal pulmonary hypertension. Arch Dis Child Fetal Neonatal Ed. (2012) 97:F223. 10.1136/adc.2009.180091 21278430

[25] Doyle LW Davis PG Morley CJ McPhee A Carlin JB. Low-dose dexamethasone facilitates extubation among chronically ventilator-dependent infants: a multicenter, international, randomized, controlled trial. Pediatrics. (2006) 117(1):75–83. 10.1542/peds.2004-2843 16396863

[26] Cherian S Morris I Evans J Kotecha S. Oxygen therapy in preterm infants. Paediatr Respir Rev. (2014) 15(2):135–41. 10.1016/j.prrv.2012.12.003 23402990

[27] Lupattelli A Barone-Adesi F Nordeng H. Association between antidepressant use in pregnancy and gestational diabetes mellitus: results from the Norwegian mother, father and child cohort study. Pharmacoepidemiol Drug Saf. (2022) 31(2):247–56. 10.1002/pds.5388 34817916

[28] Pop VJM Boekhorst MGBM Deneer R Oei G Endendijk JJ Kop WJ. Psychological distress during pregnancy and the development of pregnancy-induced hypertension: a prospective study. Psychosom Med. (2022) 84(4):446–56. 10.1097/PSY.0000000000001050 35067651

[29] Pattnaik P Palmaccio S Aschner J Eisenberg R Choi J La T Does duration off respiratory support prior to discharge from NICU predict hospital readmission among extremely low gestational age neonates? Am J Perinatol. (2021) 38(S 01):e330–7. 10.1055/s-0040-1710011 32369861

[30] Doyle LW Ranganathan S Cheong JLY. Ventilation in preterm infants and lung function at 8 years. N Engl J Med. (2017) 377(377):1601–2. 10.1056/NEJMc1711170 29045215

[31] Doyle LW. Postnatal corticosteroids to prevent or treat bronchopulmonary dysplasia. Neonatology. (2021) 118:244–51. 10.1159/000515950 33975319

[32] Yasir M Goyal A Sonthalia S. Corticosteroid adverse effects. Treasure Island: StatPearls (2021). 26 p. 30285357

[33] Walters JA Tan DJ White CJ Wood-Baker R. Different durations of corticosteroid therapy for exacerbations of chronic obstructive pulmonary disease. Cochrane Database Syst Rev. (2018) 3(3):CD006897. 10.1002/14651858.CD006897.pub4 29553157 PMC6494402

[34] DeMauro SB Jensen EA Bann CM Bell EF Hibbs AM Hintz SR Home oxygen and 2-year outcomes of preterm infants with bronchopulmonary dysplasia. Pediatrics. (2019) 143:e20182956. 10.1542/peds.2018-2956 30975699 PMC6564066

[35] Lagatta J Murthy K Zaniletti I Bourque S Engle W Rose R Home oxygen use and 1-year readmission among infants born preterm with bronchopulmonary dysplasia discharged from children's hospital neonatal intensive care units. J Pediatr. (2020) 220:40–48.e5. 10.1016/j.jpeds.2020.01.018 32093927 PMC7605365

[36] Carlo WA Vento M. Oxygen therapy for preterm infants. Clin Perinatol. (2019) 46(3):xvii–xviii. 10.1016/j.clp.2019.06.001 31345554

[37] Yoo JW Ju S Lee SJ Cho YJ Lee JD Kim HC. Geriatric nutritional risk index is associated with 30-day mortality in patients with acute respiratory distress syndrome. Medicine (Baltimore). (2020) 99(25):e20671. 10.1097/MD.0000000000020671 32569197 PMC7310893

[38] Mourani PM Kinsella JP Clermont G Kong L Perkins AM Weissfeld L Intensive care unit readmission during childhood after preterm birth with respiratory failure. J Pediatr. (2014) 164(4):749–755.e3. 10.1016/j.jpeds.2013.11.062 24388320 PMC4522939

[39] Harron K Gilbert R Cromwell D Oddie S van der Meulen J. Newborn length of stay and risk of readmission. Paediatr Perinat Epidemiol. (2017) 31:221–32. 10.1111/ppe.12359 28418622 PMC5518288

[40] Wu Y-K Lan C-C Tzeng I-S Wu C-W. The COPD-readmission (CORE) score: a novel prediction model for one-year chronic obstructive pulmonary disease readmissions. J Formos Med Assoc. (2021) 120(3):1005–13. 10.1016/j.jfma.2020.08.043 32928614

[41] Li L Yu J Wang J Zhang X Shen H Yuan X A prediction score model for risk factors of mortality in neonate with pulmonary hemorrhage: the experience of single neonatal intensive care unit in southwest China. Pediatr Pulmonol. (2008) 43:997–1003. 10.1002/ppul.20897 18785623

[42] Swets JA. Measuring the accuracy of diagnostic systems. Science. (1988) 240:1285–93. 10.1126/science.3287615 3287615

